# Lattice Vibrations and Time-Dependent Evolution of Local Phonon Modes during Exciton Formation in Conjugated Polymeric Molecules

**DOI:** 10.3390/polym13111724

**Published:** 2021-05-25

**Authors:** Yusong Zhang, Huayan Shi, Junteng Luo, Jianguo Shen, Sheng Li, Thomas F. George

**Affiliations:** 1 State Key Laboratory of Surface Physics, Department of Physics, Fudan University, Shanghai 200433, China; 16110190015@fudan.edu.cn; 2 Department of Physics, Zhejiang Normal University, Jinhua 321004, China; 202031720111@zjnu.edu.cn (H.S.); luojunteng@zjnu.cn (J.L.); shenjianguo@zjnu.cn (J.S.); 3 Department of Chemistry & Biochemistry and Physics & Astronomy, University of Missouri—St. Louis, St. Louis, MO 63121, USA

**Keywords:** phonons, excitons, conjugated polymers, dynamic simulation

## Abstract

Based on nonadiabatic molecular dynamics that integrate electronic transitions with the time-dependent phonon spectrum, this article provides a panoramic landscape of the dynamical process during the formation of photoinduced excitons in conjugated polymers. When external optical beam/pulses with intensities of 10 µJ/cm^2^ and 20 µJ/cm^2^ are utilized to excite a conjugated polymer, it is found that the electronic transition firstly triggers local lattice vibrations, which not only locally distort alternating bonds but change the phonon spectrum as well. Within the first 60 fs, the occurrence of local distortion of alternating bonds accompanies the localization of the excited-state’s electron. Up to 100 fs, both alternating bonds and the excited electronic state are well localized in the middle of the polymer chain. In the first ~200 fs, the strong lattice vibration makes a local phonon mode at 1097.7 cm^−1^ appear in the phonon spectrum. The change of electron states then induces the self-trapping effect to act on the following photoexcitation process of 1.2 ps. During the following relaxation of 1.0 ps, new local infrared phonon modes begin to occur. All of this, incorporated with the occurrence of local infrared phonon modes and localized electronic states at the end of the relaxation, results in completed exciton formation.

## 1. Introduction

In conjugated polymers, the physical picture of the exciton formation can be viewed concisely as follows: due to external optical excitation, the electron in the highest-occupied molecular orbital (HOMO) of the conjugated polymer is easily excited to the lowest-occupied molecular orbital (LUMO) [1], such that each of two electronic orbitals is then occupied by an electron. This leads to a so-called electron-hole pair, which is often called a photoinduced exciton [2,3]. To discover the mechanism of the dynamic process, previous simulations and related experimental research have been conducted based on the typical polymer MEH-PPV and its derivatives [4,5,6,7]. Furthermore, in semiconducting poly (3-hexylthiophene) (P3HT), the related studies not only demonstrate that the lattice vibration has an influence on the formation of the excited state in conjugated polymers [8], but also reveal the evolution of the exciton formation, relaxation, and radiative decay for lasers with different wavelengths [9,10,11]. Especially, it has been found out that in a solution of the conjugated polymer PBDTTT with small bandgap, the intrachain excitons play the main role during its relaxation [12].

Actually, after the electron-hole pair is formed, the following step of relaxation of the “hot” excited state is often omitted in previous research. To reveal the whole process of photoexcitation, a number of simulations have been conducted which demonstrate that, after the electron and hole are injected into a nondegenerate π–conjugated polymer, the ensuing recombination is indeed attributed to relaxation, which is caused by spontaneous one-photon processes [13]. Further, the theoretical research again points out that, during the whole dynamical process, the relaxation of exciton formation actually becomes a main part of a nonadiabatic ultrafast process [14], where the external photoexcitation triggers interband electronic transitions and self-localization, finally leading to a singlet exciton within less than 1000 fs. Further experimental research shows that highly-efficient exciton transfer is just an intrachain process rather than interchain [15]. All of this means that the development of ultrafast intrachain nonadiabatic molecular dynamics is the key step to discovering the underlying mechanism with respect to the relaxation of a hot exciton in conjugated polymeric molecules, which is the main purpose.

Besides nonadiabatic effects, during the dynamical process in a conjugated polymer, the self-trapping effect will greatly influence the lattice structure and the electronic behavior. Even two decades ago, Y. R. Shen et al. had uncovered the self-trapping effect in conjugated poly(p-phenylene vinylene) (PPV) originating from the vibrational-electronic double resonance [16], which enables a phonon to spatially track excited-state dynamics along conjugated polymeric molecules [17]. Especially at the beginning of the external photoexcitation, the structure of the conjugated polymer is easily changed by a multiple phonon process [18]. Indeed, the related experimental research again found out that, once the low-dimensional π-electrons undergo excitation, the ultrafast energy relaxation is increasingly dominated by phonon emission in which nonadiabatic electronic transitions connect to the photoinduced localization of the electron [19]. Thus, considering the self-trapping effect, it becomes the next task of this article to figure out the evolution of the phonons during the exciton formation.

Thanks to the purposes mentioned above, it becomes necessary not only to explore the underlying dynamical process with respect to the relaxation of a hot exciton, but also to clarify the contribution of phonons at the same time in conjugated polymeric molecules. Thus, in this article, nonadiabatic molecular dynamics involving an exciton will be developed where external optical excitation, coupled with the self-trapping effect of a conjugated polymer, is introduced into the nonadiabatic dynamics. Then, this article will discover the ultrafast nonadiabatic process under photoexcitation and clarify the relationship among localization, phonon modes and electronic transition dynamics.

## 2. Methodology

Based on previous research on optical excitation in conjugated polymers [20,21], a typical conjugated polymer, PPV, is selected as a model to explore the related dynamics. We employ the extended Su–Schreiffer–Heeger–Hubbard Hamiltonian, incorporating the strong electron–phonon and electron–electron interactions, and the self-trapping effect in one-dimensional conjugated polymers:

(1) $$H=H_{e}+H_{l}+H^{\prime }.$$

where *H_e_* + *H_l_* is the well-known Su–Schreiffer–Heeger Hamiltonian, which is used to describe the physical properties of conjugated polymers. The first part, *H_e_*, describes the electron-lattice interaction,

(2) $$H_{e}=-\sum_{l,s}[t_{0}-\alpha (u_{l+1}-u_{l})+{\left(-1\right)}^{l}t_{e})](a_{l+1,s}^{†}a_{l,s}+H.c.),$$

where the parameters are explained as follows: *t*_0_ (2.5–3.0 eV) is a hopping constant; *t_e_* (0.05–0.10 eV) is the Brazovskii–Kirova term; α (4.0–6.0 eV/Å) is an electron-lattice interaction constant; and $a_{l,s}^{†}/a_{l,s}$ is the electron creation/annihilation operator. *H_l_* is for the kinetic energy and elastic potential energy of the polymer lattice,

(3) $$H_{l}=\frac{M}{2}\sum_{l}{\left(\overset{\cdot }{u_{l}}\right)}^{2}+\frac{K}{2}\sum_{l}{\left(u_{l+1}-u_{l}\right)}^{2},$$

where *K* (20–30 eV/Å^2^) is an elastic constant, and *M* is the group mass. *H′* represents the electron–electron interaction, which is actually the extended Hubbard electron interaction:

(4) $$H^{\prime }=U\sum_{l}n_{l,↑}n_{l,↓}+V\sum_{l,s,s^{\prime }}n_{l,s}n_{l+1,s^{\prime }}.$$

The parameter *U* (2.0–5.0 eV) is for the on-site Coulomb interaction, while *V* (0.5–2.0 eV) is for the nearest–neighbor Coulomb interaction. For convenience of computation, *H′* is treated within the Hartree–Fock approximation as

(5) $$H^{\prime }=\sum_{l,s}\{U\left(\sum_{\mu }^{occ}{\left|Z_{l,\mu }^{-s}\right|}^{2}-\frac{1}{2}\right)+V\left[\sum_{s^{\prime }}\left(\sum_{\mu }^{occ}{\left|Z_{l+1,\mu }^{-s^{\prime }}\right|}^{2}+\sum_{\mu }^{occ}{\left|Z_{l+1,\mu }^{-s^{\prime }}\right|}^{2}-2\right)\right]\}c_{l,s}^{†}c_{l,s}-\sum_{l,s}\left(V\sum_{\mu }^{occ}Z_{l,\mu }^{s}Z_{l+1,\mu }^{s}\right)c_{l,s}^{†}c_{l,s}+H.c.\;\;,$$

where *occ* is the occupation number.

For nonadiabatic dynamics, the time-dependent Schrödinger equation is employed directly:

(6) $$i\hbar \frac{\partial }{\partial t}\Psi =H\Psi$$

if the initial electronic wavefunction is $\Psi (t_{i})$ and the final is $\Psi (t_{f})$, the time-independent Hamiltonian during a very short span of time ($t_{f}-t_{i}$) is

(7) $$\Psi (t_{f})=\exp\{-\frac{iH}{\hbar }(t_{f}-t_{i})\}\Psi (t_{i})$$

beginning with *t*_1_, *t*_2_ (until *t_N_*), the electronic wavefunction of the later time is the time evolutional result of the wavefunction at the earlier time. If the interval is quite short, such as 0.01 fs, the wavefunction at any time can be acquired. Then, the electronic wavefunction at any time can be calculated by a linear combination:

(8) $$\Psi (t)=\sum_{\mu }c_{\mu }(t)\Phi_{\mu }\;\;.$$

here $\Phi_{\mu }$ is an eigenfunction, where due to the orthonormality of the eigenfunctions, the expansion coefficients can be written as

(9) $$c_{\mu }(t)=\langle \Phi_{\mu }|\Psi (t)\rangle \;\;.$$

If an optical field with a certain frequency *ω* and energy density *ρ* can induce an electron transition between energy levels Γ*_u_* and Γ*_d_*, the transition rate can be expressed as

(10) $$W_{d\to u}=\frac{4\pi^{2}}{3\hbar^{2}}p^{2}\rho (w)\delta (w-\frac{E_{u}-E_{d}}{\hbar })$$

The spontaneous transition rate γ*_ud_* between these two states is written as

(11) $$\gamma_{u\to d}=\frac{4(E_{u}-E_{d}{)}^{3}}{3\hbar^{4}c^{3}}p^{2}$$

As for the lattice vibrations, the matrix is introduced to obtain the phonon modes. Here, given the static lattice configuration at site *n* as $\phi_{n}^{0}$, its perturbation can be written as

(12) $$\phi_{n}^{}(t)=\phi_{n}^{0}+\phi_{n}^{\prime }(t).$$

Based on second-order perturbation theory in the calculation of the energy, the phonon mode can be determined:

(13) $$H(\{\phi_{n}\})=E_{0}+E_{s}\sum_{m}^{N}A_{m}(\{\phi_{n}\})\phi_{m}^{\prime }+\frac{1}{2}\sum_{m,n}^{N}B_{m,n}(\{\phi_{n}^{0}\})\phi_{n}^{\prime }\phi_{m}^{\prime }$$

(14) $$B_{m,n}=k[(\delta_{m,n}+\delta_{m,n+1})(1-\delta_{m,N})+(\delta_{m,n}+\delta_{m,n-1})(1-\delta_{n,1})]+2\alpha^{2}{\left(-1\right)}^{m+n}\sum_{\mu ,\nu (\neq \mu )}\frac{C_{\mu ,\nu }^{m}C_{\mu ,\nu }^{n}}{\varepsilon_{\mu }^{0}-\varepsilon_{\nu }^{0}}$$

here, *N* is the total number of lattice sites; *ε* is the static eigenenergy of the electron; and the parameter $C_{\mu ,\nu }^{m}$ is

(15) $$C_{\mu ,\nu }^{m}=(1-\delta_{m,N})(Z_{\mu ,m+1,s}^{0}Z_{\nu ,m,s}^{0}+Z_{\mu ,m,s}^{0}Z_{\nu ,m+1,s}^{0})-(1-\delta_{m,1})(Z_{\mu ,m,s}^{0}Z_{\nu ,m-1,s}^{0}+Z_{\mu ,m-1,s}^{0}Z_{\nu ,m,s}^{0}),$$

where $Z_{\mu ,m,s}^{0}$ is the corresponding eigenstate of the electron in energy level *μ* at lattice site *m* with spin value *s*, and $\delta_{m,n}$ is the Kronecker delta. After diagonalizing the B matrix, the eigenfrequency and eigenvector of the phonon modes can be obtained. As a result, the excitation of phonon modes during the formation process of the hot exciton can be well described.

## 3. Results and Discussion

The occurrence of the electron-hole pair can be briefly described as follows: when a conjugated polymer is excited by an external beam whose energy just covers the bandgap between the HOMO and LUMO, the HOMO electron starts moving to the LUMO. Ultimately, the LUMO obtains an extra electron and the HOMO a hole, yielding an electron-hole pair.

Tracing back to previous articles [10,11,12], the experimental research finds that, when the conjugated polymer is excited by a laser beam with an intensity ranging from 6 to 40 μJ/cm^2^, exciton formation is seen to finish in around 1ps while the electron is localized within 100 fs. Considering the fact that electron localization occurs with the formation of the exciton, it raises the question as to whether the electron localization just means the formation of the exciton.

To resolve this puzzle, we here applied nonadiabatic molecular dynamics developed on the basis of that used in [20,21] to reveal the whole relaxation of intrachain exciton formation as mentioned in reference [14]. The developed nonadiabatic molecular dynamics not only couples with external optical excitation leading to the self-trapping effect of a conjugated polymer, but also is able to illustrate the evolution of the phonon spectrum and phonon modes from Equations (12)–(15) in the section called Methodology.

### 3.1. Electron Localization and Photoinduced Exciton Formation

**Figure 1:**
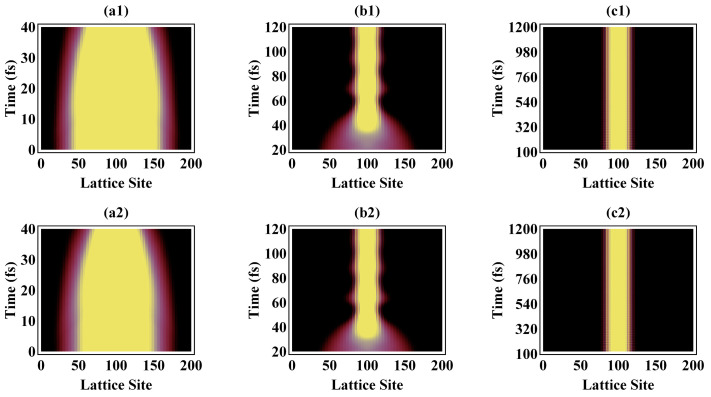
Spatial distribution of the electron over the lattice sites under an external optical field of 10 μJ/cm^2^ (**top**) and 20 μJ/cm^2^ (**bottom**).

Firstly, let us choose an optical field of 10 μJ/cm^2^ and 20 μJ/cm^2^ upon PPV to simulate the whole ultrafast dynamics of the photoexcited process. Figure 1 depicts the temporal evolution of the electronic state along the polymer chain for the external optical field of 10 μJ/cm^2^ (top) and 20 μJ/cm^2^ (bottom). In the first 30 fs, as shown in Figure 1a1,a2, the electronic state is extensive, covering 150 units of the entire polymer chain of 200 units. After 40 fs, the spatial distribution of the electron starts shrinking and finally localizes at the center of the PPV chain until around 60–80 fs, which is in Figure 1b1,b2. And then, from 80 to 100 fs, as shown in Figure 1c1,c2, the spatial localization of the electronic state results in sharp stabilization, where the photoexcitation with a field of intensity 10 μJ/cm^2^ takes 60 fs for the localization of the electron, consistent with the previous experimental research [10,11,12].

**Figure 2:**
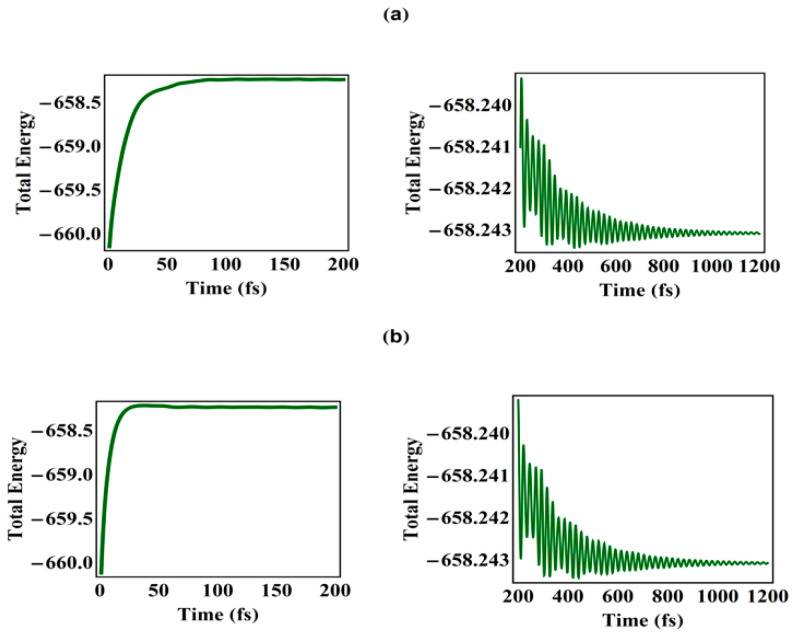
Total energy of the polymer chain system under an external optical field with intensity (**a**) 10 μJ/cm^2^ and (**b**) 20 μJ/cm^2^.

From previous experimental research, it is found that, under photoexcitation by a field of intensity 6 μJ/cm^2^, it takes 60 fs for the localization of the electron in contrast to 1.0 ps for the exciton formation [10,11,12,15]. As for the cases presented in Figure 1, the simulation of spatial distribution of electron in the polymeric chain demonstrates that, although the field intensity is increased from 10 to 20 μJ/cm^2^, electron localization still occurs within ~60 fs, which is in keeping with recent experiments that the electron localization takes place within 100 fs [11]. To clarify this, Figure 2 illustrates the relaxation of the total energy (electron and lattice energies) with respect to time until 1.2 ps. It is shown that the total energy of PPV significantly increases in the first 75 fs (Figure 2a, 10 μJ/cm^2^) and 40 fs (Figure 2b, 20 μJ/cm^2^) as it absorbs energy from the external optical field. Just within this time span, the electron localization has been completed. Since then, the PPV polymeric molecule, under an external optical field of 10 μJ/cm^2^ and 20 μJ/cm^2^, has been undergoing the relaxation process, while its total energy has been oscillating until 1 ps, finally producing the localized exciton state at 1.2 ps. Mostly, the evolution of the total energy of the polymer chain demonstrates that the newly resultant exciton has to undergo relaxation of ~1.0 ps to anneal the “hot” exciton, and finally forming the exciton, which is consistent with previous experimental research [10,11,12,15].

### 3.2. Temporal Evolution of Alternating Bonds

In a conjugated polymer, the electron distribution in a molecular orbital can change the structure of the molecular skeleton. At the same time, the change of lattice structure alters the structure of the electron spectrum, thus leading to a continuous mutual influence of both the lattice structure and electron. The effect described above is the characteristic “self-trapping effect” of conjugated polymers. Because alternating bonds are generally regarded as characterizing the lattice structure of the conjugated polymer, the alternating bonds is then selected to illustrate the evolution of the polymer lattice.

**Figure 3:**
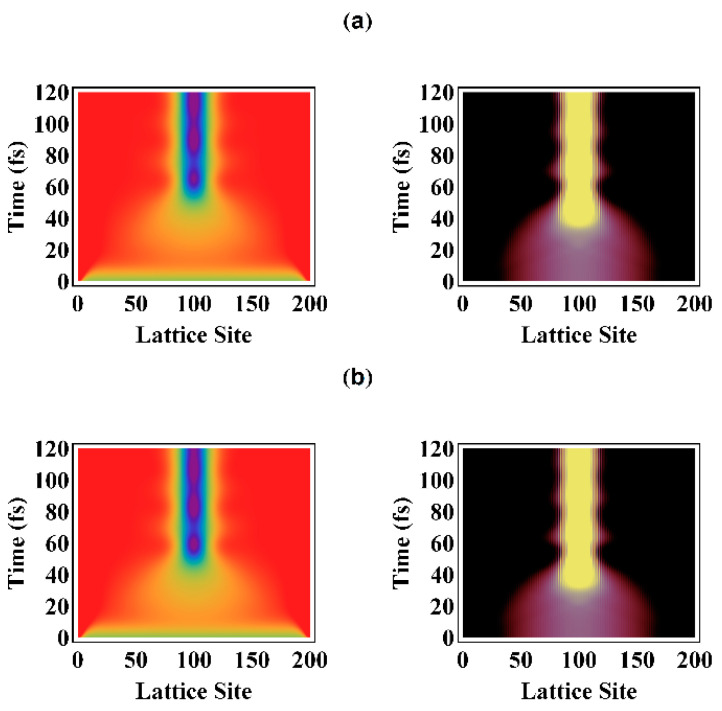
Under optical fields of intensity (**a**) 10 μJ/cm^2^ and (**b**) 20 μJ/cm^2^, evolution of alternating bonds within the first 120 fs (**left**), and spatial distribution of the electron distribution (**right**) over the lattice sites within the first 120 fs.

We first introduce the lattice configuration *ϕ_n_* to describe the profiles of the alternating bonds. If the displacement of every group in the PPV chain is *u_n_*, the lattice configuration describing alternating bonds is *ϕ_n_* = (−1)*^n^u_n_*. Comparing two temporal evolutions for the external optical field with the intensities of 10 μJ/cm^2^ and 20 μJ/cm^2^, it is found that the localized distortion of the alternating bonds appears as the localization of the electron distribution, as shown in Figure 3. For the first 20 fs of photoexcitation, as shown in Figure 3a,b (left), the alternating bonds have a slightly visible distortion. As the alternating bonds in a conjugated polymer have been locally distorted, the spatial distribution of the electron is also localized as Figure 3a,b (right). At ~60 fs, the local distortion of alternating bond becomes distinct. By 100 fs and beyond, the local lattice distortion is stabilized and prominent as shown in Figure 3a,b (left). Mostly, it can be seen in Figure 3 that within the first 60 fs, the local distortion of alternating bonds was at ~10 fs after the localization of the excited-state’s electron.

**Figure 4:**
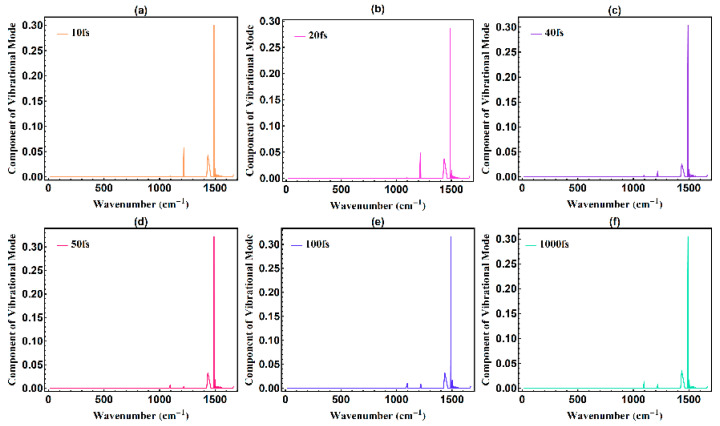
Phonon spectrum at 10, 20, 40, 50, 100, and 1000 fs during excitation by an optical field of intensity of 20 µJ/cm^2^.

As mentioned in the introduction, the self-trapping effect of the conjugated polymer enables phonons to spatially affect the excited-state dynamics [16,17]; thus, we utilized the developed nonadiabatic molecular dynamics to show the evolution of phonon modes and their contribution to the exciton formation. From the demonstration above, the lattice vibrations are tightly associated with the evolution of the electronic state. We focus our attention on the phonon modes when the conjugated polymer undergoes excitation from the optical field with 20 µJ/cm^2^. During the optical excitation from the beginning until 1 ps, the phonon spectrum changes accordingly, where Figure 4 illustrates the phonon spectra at the six different times of 10, 20, 40, 50, 100, and 1000 fs. At the beginning of the photoexcitation, there exists four peaks at 1218.3 1434.2, 1490.3, and 1501.3 cm^−1^, and another peak at 1097.7 cm^−1^ then occurring in the phonon spectrum.

**Figure 5:**
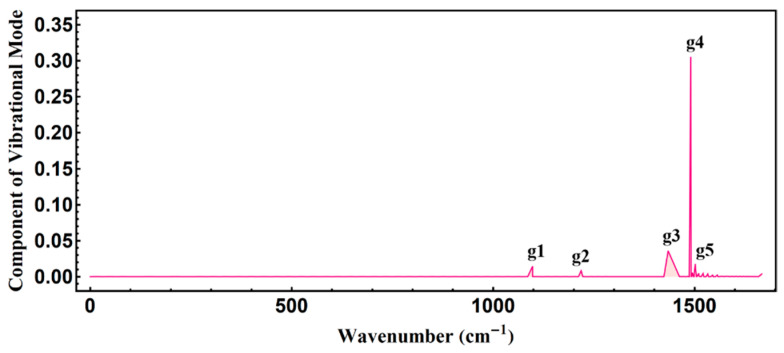
Phonon spectra of the exciton under pumping by an external laser of 20 µJ/cm^2^.

**Figure 6:**
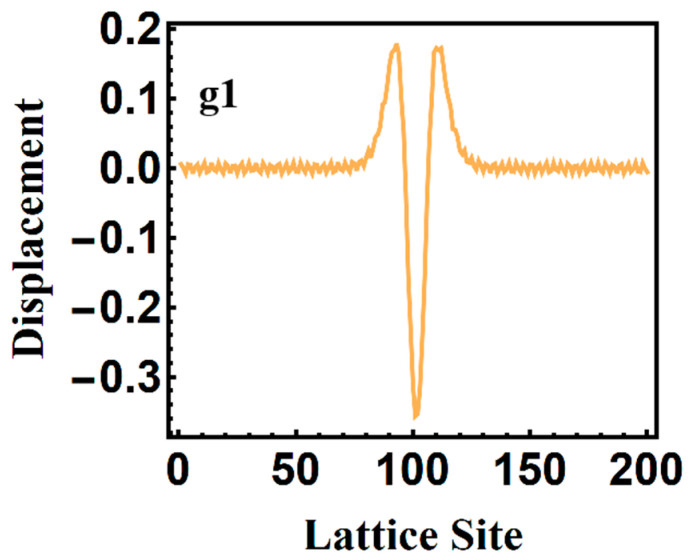
Local phonon mode at 1097.7 cm^−1^ under photoexcitation by an external optical field of 20 µJ/cm^2^.

Here, from Figure 4, it can be found that multiple phonon emission occurs along with the whole ultrafast energy relaxation. This ultrafast multiple phonon emission process is in accordance with previous experimental research [18]. Also, the multiple phonon emission process is dominated by four phonon modes of 1218.3, 1434.2, 1490.3, and 1501.3 cm^−1^, which exactly reflects the facts found in previous experimental research, that the ultrafast energy relaxation is increasingly dominated by phonon emission during the nonadiabatic electronic transitions [19]. The appearance of the new peaks in the phonon spectrum, after photoexcitation by an optical field of 20 µJ/cm^2^, indicates the change of alternating bonds in the conjugated polymer. After relaxation at 1200 fs, five peaks appear in the phonon spectrum, as seen in Figure 5. The new peak of 1097.7 cm^−1^ in the phonon spectrum is marked with g1, and its characteristic phonon mode is illustrated in Figure 6.

**Figure 7:**
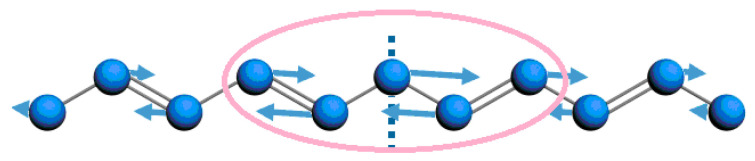
Vibrational configuration of the local phonon mode at 1097.7 cm^−1^ under photoexcitation by an external optical field of 20 µJ/cm^2^.

With regard to the vibrational configuration {*ϕ_n_*} of the local phonon mode at 1097.7 cm^−1^ as depicted in Figure 6, we can easily obtain the displacement of every group, *ϕ_n_* = (−1)*^n^u_n_*, where *u_n_* is the actual displacement in the PPV chain and *ϕ_n_* is the lattice configuration (also called alternating bonds). Based on the local phonon mode at 1097.7 cm^−1^ of Figure 6, the schematic figure of the vibration of groups in the PPV chain can be drawn as Figure 7, where the circle of pink color means the region of localized lattice distortion. Mostly, the obtained local phonon mode at 1097.7 cm^−1^ based on our Hamiltonian describing PPVs contributes to the formation of the exciton after annealing of the “hot” exciton, which also can be reflected by the recently observed 2^1^A_g_^−^ exciton transport in one-dimensional blue conjugated polymerized polydiacetylenes without being twisted [22]. Apparently, from Figure 6, the new localized mode possesses even parity. Thus, the new phonon mode is an infrared mode.

Thus, the underlying mechanism can be discovered as follows: after the polymeric molecule PPV absorbs energy from an external optical field, the electron transits from the molecular orbital HOMO to LUMO. Within the first 40 fs of photoexcitation, the total energy rapidly increases. The change of electron states then induces the self-trapping effect to act on the following photoexcitation process of 1.2 ps. In the first ~200 fs, the strong lattice vibration makes a local phonon mode at 1097.7 cm^−1^ appear in the phonon spectrum. Then, the occurrence of this mode in turn triggers the electron localization immediately. Due to the mass of the electron being smaller than that of the units of PPV, electron spatial localization is more easily formed than lattice localization, which also leads to localized distortion of the alternating bonds to occur slightly after electron spatial localization (10~20 fs). After that, the total energy continues to oscillate until around 1.0 ps, which is regarded as relaxation of the change of electron states.

## 4. Summary

In summary, this article employs nonadiabatic molecular dynamics based on what is used in [20,21]. This not only couples with external optical excitation, leading to the self-trapping effect of a conjugated polymer, but also is able to illustrate the evolution of the phonon spectrum and phonon modes, to reveal the whole relaxation of the intrachain exciton formation in reference [14]. We then choose an optical field of 10 μJ/cm^2^ and 20 μJ/cm^2^ upon PPV to simulate the whole ultrafast dynamics of the photoexcited process. It is observed that the PPV polymeric molecule, under an external optical field of 10 μJ/cm^2^ and 20 μJ/cm^2^, undergoes a relaxation process, while its total energy oscillates until 1 ps, finally producing the localized exciton state at 1.2 ps. The newly resultant exciton has to undergo relaxation of over 1.0 ps to anneal the “hot” exciton, finally forming the exciton, which is consistent with previous experimental research [10,11,12,15]. As for the self-trapping effect of the conjugated polymer mentioned in [16,17], multiple phonon emission occurs along with the whole ultrafast energy relaxation. Also, the multiple phonon emission process is dominated by four phonon modes of 1218.3, 1434.2, 1490.3, and 1501.3 cm^−1^, all of which exactly reflects what is found in previous experimental research [17,18,19]. Within the first 40 fs of photoexcitation, the total energy rapidly increases. The change of electronic states then induces the self-trapping effect to act on the following photoexcitation process: the strong lattice vibration causes a local phonon mode to appear at 1097.7 cm^−1^ in the phonon spectrum. Additionally, this mode, based on our Hamiltonian describing PPVs, contributes to the ultimate formation of the exciton after annealing of the “hot” exciton, which also can be reflected by the recently observed 2^1^A_g_^−^ exciton transport in one-dimensional blue conjugated polymerized polydiacetylenes (without being twisted) [22]. The new localized mode possesses even parity, which is an infrared phonon mode. After relaxation by 1.2 ps, all of this, incorporated with the local infrared phonon mode at the end of the relaxation, completes the formation of the exciton.
